# Temporal Patterns of Larval Fish Occurrence in a Large Subtropical River

**DOI:** 10.1371/journal.pone.0146441

**Published:** 2016-01-13

**Authors:** Fangmin Shuai, Xinhui Li, Yuefei Li, Jie Li, Jiping Yang, Sovan Lek

**Affiliations:** 1 Pearl River Fisheries Research Institute, CAFS, Guangzhou, Guangdong, China; 2 Experimental Station for Scientific Observation on Fishery Resources and Environment in the Middle and Lower Reaches of Pearl River, Ministry of Agriculture, Guangdong, China; 3 Key Laboratory of the Conservation and Ecological Restoration of Fishery Resource in the Pearl River, Guangzhou, Guangdong, China; 4 Université de Toulouse—Paul Sabatier, 118 route de Narbonne, Toulouse Cedex, France; University of Sydney, AUSTRALIA

## Abstract

Knowledge of temporal patterns of larval fish occurrence is limited in south China, despite its ecological importance. This research examines the annual and seasonal patterns of fish larval presence in the large subtropical Pearl River. Data is based on samples collected every two days, from 2006 to 2013. In total, 45 taxa representing 13 families and eight orders were sampled. The dominant larval family was Cyprinidae, accounting for 27 taxa. *Squaliobarbus curriculus* was the most abundant species, followed by *Megalobrama terminalis*, *Xenocypris davidi*, *Cirrhinus molitorella*, *Hemiculter leuscisculus* and *Squalidus argentatus*. Fish larvae abundances varied significantly throughout the seasons (multivariate analyses: Cluster, SIMPROF and ANOSIM). The greatest numbers occurred between May and September, peaking from June through August, which corresponds to the reproductive season. In this study, redundancy analysis was used to describe the relationship between fish larval abundance and associated environmental factors. Mean water temperature, river discharge, atmospheric pressure, maximum temperature and precipitation play important roles in larval occurrence patterns. According to seasonal variations, fish larvae occurrence is mainly affected by water temperature. It was also noted that the occurrence of *Salanx reevesii* and *Cyprinus carpio* larvae is associated with higher dissolved oxygen (DO) concentrations, higher atmospheric pressure and lower water temperatures which occur in the spring. On the other hand, *M*. *terminalis*, *X*. *davidi*, and *C*. *molitorella* are associated with high precipitation, high river discharge, low atmospheric pressure and low DO concentrations which featured during the summer months. *S*. *curriculus* also peaks in the summer and is associated with peak water temperatures and minimum NH_3_–N concentrations. *Rhinogobius giurinus* occur when higher atmospheric pressure, lower precipitation and lower river discharges occur in the autumn. Dominant fish species stagger their spawning period to avoid intraspecific competition for food resources during early life stages; a coexistence strategy to some extent. This research outlines the environmental requirements for successful spawning for different fish species. Understanding processes such as those outlined in this research paper is the basis of conservation of fish community diversity which is a critical resource to a successful sustainable fishery in the Pearl River.

## Introduction

It is well known that in order to maintain fishery resources it is very important to conserve biodiversity and ecosystem functions. However, global fishery resources are in decline due to many man-induced stressors such as overfishing, dam construction, biological invasions and climate change [1–2]. Fishing moratoria, such as no fishing during spawning seasons to restore stock, has become a major conservation tool [3–4]. There is however, a dearth of information regarding fishes spawning patterns. Conservation planning decisions are thus often made without actually quantifying the benefits for the ecological processes they intend to conserve. A paucity of knowledge of temporal patterns of the occurrence of juvenile fish species can have dramatic adverse consequences on the quality and effectiveness of fishing moratoria. It is known that the reproductive strategies of fishes are as a result of long-term natural evolution, but the actual timing of larval production can determine the success of recruitment [5–6]. Variations in the abundance of larvae within a season can be as important as inter-annual changes [7]; such patterns need to be ascertained to provide accurate planning procedures to support healthy, well-managed fisheries. Furthermore, in order to reliably evaluate the status of fish resources in a particular area, it is important to understand and take into account the temporal characteristics of fish activities [8].

Since fish larval abundance and species composition largely reflect the population and dynamics of the adult fish, larval sampling surveys are an effective means to obtain information on populations compared to adult fish analysis [9–13]. Collecting fish larvae data provides reliable information on the spawning seasons and population dynamics of fish in aquatic ecosystems [13–14]. Ichthyoplankton are greatly influenced by their local environments, including any meteorological events such as precipitation, freshwater discharges [15] and temperature [16], which can all lead to shifts in the community structure and changes in species interactions. The sensitivity of the early life stages of fish potentially makes fish larvae an important resource in the study of fish ecology. The study of these early life stages, such as larvae and juveniles, may also be important harbingers of phase shifts in ecosystem dynamics [17].

In recent years, there has been increasing interest in the occurrence patterns of fish larvae. Temporal patterns have been central to numerous studies throughout global oceanic and freshwater ecosystems [18–22]. The fact that research focuses on why patterns occur in certain periods has important practical significance [23], both for the protection and utilization of fishery resources.

The Pearl River, which flows into the South China Sea, is a large subtropical river; it is the longest in south China, 2,400 kilometres. The Pearl River, with a warm and humid climate, supports 381 fish species, i.e. 262 freshwater species and 119 estuarine species, including five exotic species [24]. The Pearl River has a high species diversity and is considered to be of great significance in human consumption and aquatic biodiversity. The Pearl River has experienced numerous man-induced disturbances since the 1950s, including dam construction to create reservoirs, pollution, overfishing, and also channel modifications in the main stretch of the river. Such disturbances are known to negatively impact the biotic and abiotic components of riverine ecosystems [25]. Since the 1960s, the abundance of several fish species has declined or indeed some have been totally extirpated [26]. Surveys in the Pearl River from 2006–2008 found relationships between high larval densities and prevailing hydrological characteristics [25]. There are no published records of other studies on fish larvae in the Pearl River. Local temporal patterns of juvenile fish species occurrence have also not been investigated. Such studies are important both for developing informed conservation decisions and understanding the status of fish resources in the Pearl River.

The aim of this study is to describe the annual and seasonal patterns of fish larval occurrence. The underlying influences relating to those patterns will also be determined by modeling the relationships between fish larvae and environmental factors, using redundancy analysis (RDA).

## Materials and Methods

### Study site

**Fig 1:**
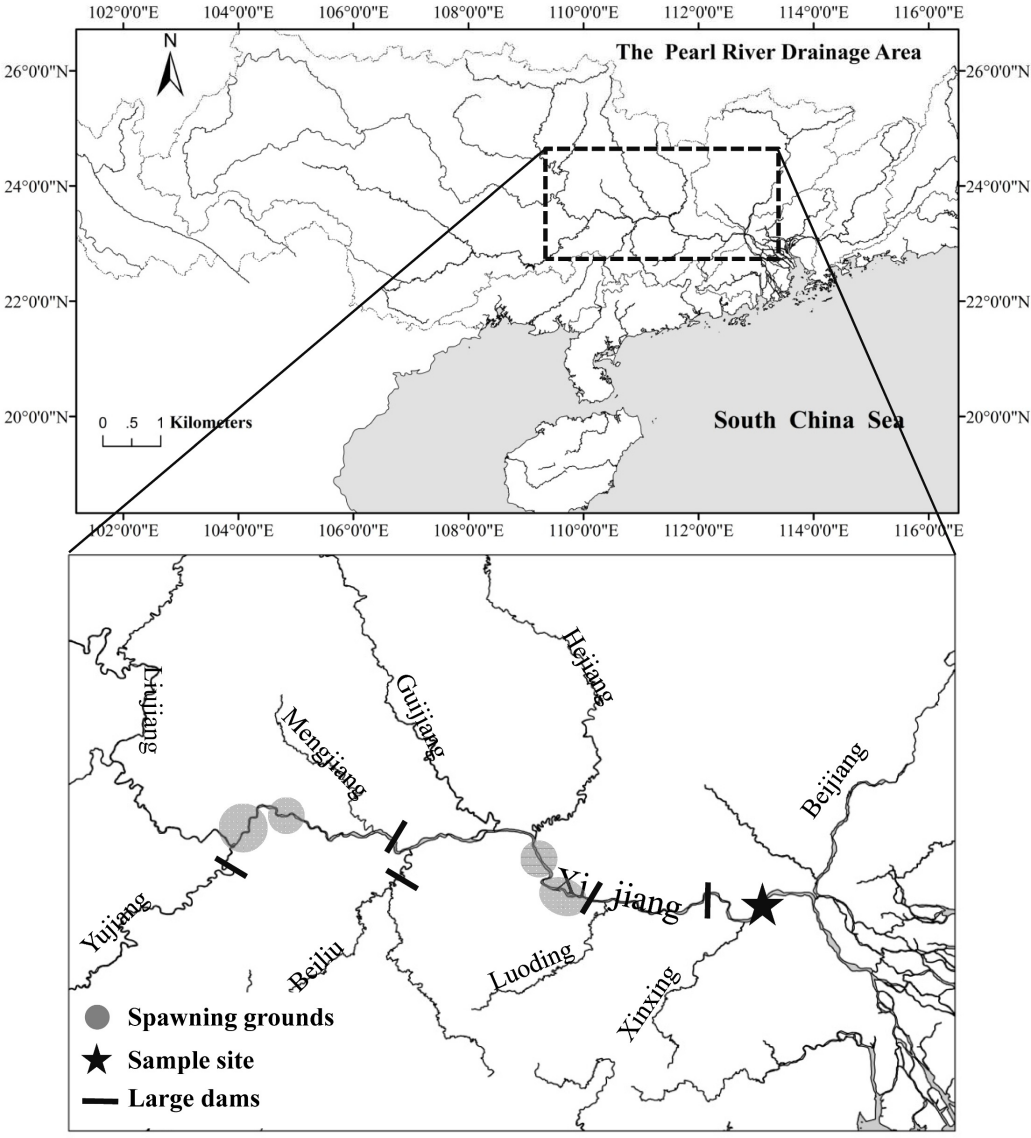
Study site.

The study site (23°2'40"N, 112°27'5"E) was located in the Zhaoqing section of the Pearl River, approx. 100 km upstream of the Pearl River estuary (Fig 1). The spawning grounds of most fishes are distributed in the middle and upper regions of the Xi Jiang River i.e. the West River—one of three major tributaries of the Pearl River (Fig 1). The Zhaoqing section of the Xi Jiang River is an important area for collecting larvae, since it is the location where drifting eggs and larvae enter the Pearl River Delta network, at the beginning of the flood season [26]. To gain insight into the ecosystem equilibrium and fish stock composition, as well as temporal patterns of fish larval occurrence, planktonic fish larvae were monitored in the Pearl River from the year 2006 to date.

### Ethics statement

Research involving animal experimentation has been approved by the Pearl River Fishery Management Council (PRFMC) and the Pearl River Fisheries Research Institute (PRFRI, authorization number 45541566–7). All experiments performed on animals in this study complied with China regulations regarding the use and care of laboratory animals. All analyses were performed to minimize suffering. This work was approved by China Wildlife Conservation Association (CWCA). All sampling procedures were approved as part of obtaining the field permit by Experimental Station for Scientific Observation on Fishery Resources and Environment in the Middle and Lower Reaches of Pearl River, Ministry of Agriculture of China.

### Data collection and sampling

Larval samples were collected using a Jiang net (total length 2 m; rectangular iron opening/mouth 1.0 m × 1.5 m and a mesh net size of 0.5 mm attached to a 0.8 m × 0.4 m × 0.4 m filter collection bucket). Fishing gear was consistently deployed 10 m from the shoreline. Samples were collected three times a day, between the hours of 06:00–08:00 h, 13:00–15:00 h, and 19:00–21:00 h, and every 2 days throughout the year [27]. A flow meter was mounted in the mouth of the net to estimate the volume of water filtered. This value was used to calculate larval density (number m^−3^). Collected larvae were immediately fixed with 5% formalin. Fish larvae were identified to the lowest taxon possible according to Liang [28], Yi et al. [29], and Cao et al. [30]. Relative position of the dorsal and anal fins, spines, fin rays, and vertebrae counts were used to identify the fish larvae.

Data concerning river discharge and average water temperatures were provided by the Pearl River Water Conservancy Commission. Maximum and minimum temperature, atmospheric pressure and precipitation were collected from http://www.weatheronline.co. The COD, NH_3_–N, dissolved oxygen (DO) concentrations and pH were provided by China National Environmental Monitoring Centre. These environmental datasets were used to describe the seasonal changes at the study site, and represent the approximate instream chemical conditions for the fish larvae. All environmental data were organized on a 2-day basis to coordinate with the larval collecting schedule.

In order to reduce deviation, every 2 day datasets were averaged into weekly datasets each month in each year, before data analysis.

### Data analysis

In order to establish and portray potential patterns, principal component analysis (PCA) was used. Months have been grouped into three seasons according to the weather conditions in South China. This area is, characterized by a warm and humid climate, with an average temperature of 23°C and has no obvious winter season (therefore omitted). The three seasons outlined are spring (February, March, April), summer (May, June, July, August and September) and autumn (October, November, December and January). Abundance patterns were analyzed each month based on the weekly dataset.

Annual and seasonal patterns of species occurrence was examined by cluster analysis and ordination using the mean abundance each month and each year. In this study, only numerically abundant species were tested, i.e. only those with an abundance of more than 0.01(ind∙m^–3^) to avoid any spurious effects of groups of rare species [31]. Two-dimensional heat dendrograms were produced to examine temporal group relationships; sets of samples were clustered together based on the similarity of their larval occurrence patterns [32]. The heatmap re-arranges the rows and columns of datasets so that similar rows, and similar columns, are grouped together, and their similarity represented by a dendrogram. Numerical values are displayed using colours. The heatmap was suitable as an initial exploratory tool of collected data. Cluster analysis on the other hand is known to have more advanced clustering functions, and produces more precise clustering of variables [33]. In this study, the heatmap cluster analysis method was used to analyze the annual and seasonal occurrence patterns of fish larvae. Data per year and per month were firstly reduced by selecting the annual mean abundance and monthly mean abundance before carrying out the analysis. Larvae matrix was selected for columns, and time as rows.

In order to guard against the interpretation of sample patterns that could have been obtained by chance, statistical testing was needed. The ‘similarity profile’ (SIMPROF) analysis was selected, which tested for the presence of sample groups in a priori unstructured set of samples [34–35]. The SIMPROF test is the biotic similarities from a group of a priori unstructured samples, ordered from smallest to largest and plotted against their rank (the similarity profile). This profile is compared with that expected under a simple null hypothesis of no meaningful structure within that group. Repeated application of this test generates a stopping rule for a posteriori division of the samples into ever smaller subgroups, as in hierarchical cluster analysis. Independent permutation tests (recalculated randomly 1000 times) were performed to generate the expected distribution of the data [36–37]. To reject the null hypothesis, a stringent P value of 0.01 was set.

The one-way non-parametric analysis of similarity (ANOSIM) combined with a randomization test for significance [34], was used to test the significance of observed differences between year groups and seasons in fish assemblage structure. ANOSIM is a nonparametric permutation procedure that tests whether differences in dissimilarity between groups exceeds differences within groups [38]. R-statistic values for pair-wise comparisons, provided by ANOSIM were used. The ANOSIMR statistics (i.e., global R) fall between −1 and 1 with R = 0 indicating completely random grouping, while R = 1 indicates that all replicates of a group are more similar to each other, than to any other groups [39]. Species showing ratios of average similarity or dissimilarity to standard deviation (r)˃2 were considered as typical (in similarity analyses) or discriminatory (in dissimilarity analyses) [40].

Asymmetric canonical analysis has become an instrument of choice for ecologists who want to relate a data table (Y) of response variables (such as species abundances) to a second data table (X) of explanatory variables (often environmental factors). However, when modelling time with a principal coordinate of neighbourhood matrices (PCNM) approach, the RDA consistently outperformed the other statistical tests using all data [41–42]. RDA is widely used by ecologists and combines regression and principal component analysis (PCA). It can be used to model the relationship between the response variables and explanatory variables by means of multiple linear regressions and eigenvalue decomposition of fitted values [43]. ANOVA permutation tests (replicated randomly 1000 times) were performed to evaluate the model's performance and significance of constraints. In this study, RDA was applied to determine how environmental variables affect the occurrence pattern and to find out which environmental variables were most related to species occurrence, based on abundance.

Finally, to further examine the interaction between years and seasons in the study period, a two-factor orthogonal design method (ANOVA combined with linear regression models), was used to test the interaction of months over years, or years over months.

Larvae data underwent Hellinger transformation and the environmental data were log_e_(y+1) transformed before analysis. The Hellinger transformation is an application recommended for clustering or ordination of species abundance data. For linear ordination, the Hellinger distance offers a better compromise between linearity and resolution than the chi-square metric and the chi-square distance [37, 44].

All analyses were performed using R Statistical Software (R Core Development Team, 2011).

## Results

### Environmental factors

**Fig 2:**
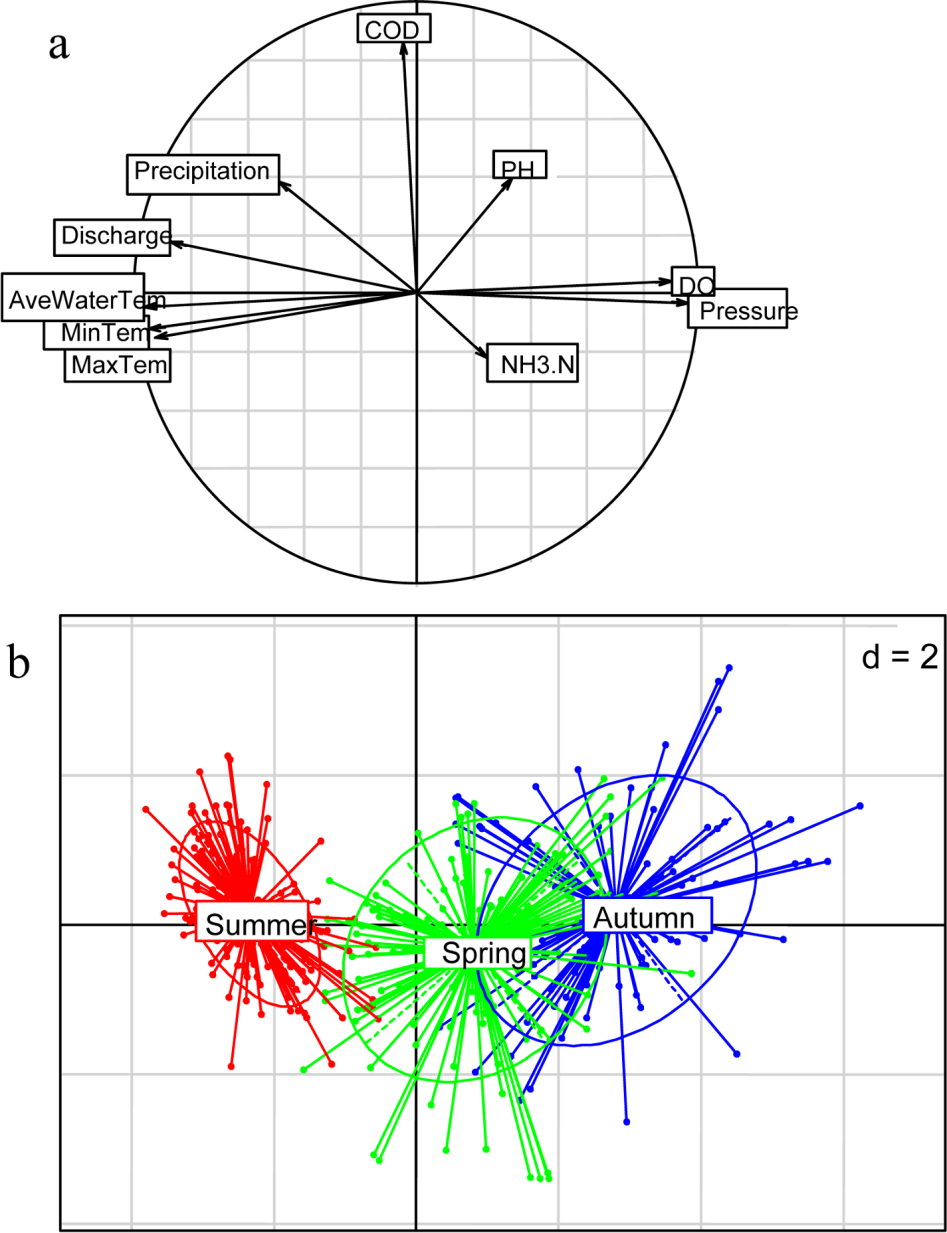
Temporal variations in environmental factors in Pearl River from January 2006 to December 2013. (a) Correlations between the environmental variables and their scores on axes 1 and 2, the circle of radius 1 represents the maximum length of a partial standardized axis. (b) Mutlivariate analyses of environmental variables using a scatter diagram by seasons.

Temporal variations of ten environmental factors are shown in Fig 2. Average water temperature, maximum temperature, minimum temperature, river discharge and precipitation were highly correlated with each other, and negatively associated with DO and pressure. They are linked to axis 1 which accounts for 56% of total variance. Chemical oxygen demand (COD) is linked to axis 2, explaining 12% of total variance. All environmental factors showed significant seasonal gradients along axis 1, opposing the summer season characterized by high river discharge, high precipitation and high temperature to the low seasons (spring and autumn) with low temperatures, high pressure and dissolved oxygen concentrations and high atmospheric pressure. The inter-annual variability of the environmental factors was not obvious.

### Species composition

A total of 43,800 bottles of samples were collected, comprising 45 taxa, representing 13 families and eight orders (Table 1). Most larval samples were at the one-chamber, gas-bladder stage, with body lengths between 5.2 and 18 mm. Total abundance was lowest in 2011 with only 16.23×10^10^ individuals (ind.) estimated. Highest numbers were recorded in 2013 with 61.76×10^10^ind. The Cyprinidae family, represented by 27 taxa, accounted for almost half of the total fish species captured. *S*. *curriculus* (Squai, Barbel chub) dominated the catch every year.

**Table 1:** Species composition and mean density (No. of larvae per m^3^) of the fish larvae collected in the Pearl River during 2006–2013. Data for each year are averaged for all days. “+” indicates rare species; N = the total number of taxa collected in each year (N includes rare taxa removed before analysis; bracket contents are abbreviations used for species.

Taxon	2006	2007	2008	2009	2010	2011	2012	2013
**CLUPEIFORMES**								
Engraulidae								
*Coilia grayii* (Coil)	+	0.01	0.01	0.03	+	+	0.06	0.01
**BELONIFORMES**								
Hemirhamphidae (Hemi)								
*Rhynchorhamphus georgii* (Rhyn)	+	+	+	+	+	+	+	+
**CYPRINIFORMES**								
Cyprinidae								
*Acrossocheilus beijiangensis* (Acro)	+	+	+	+	+	+	+	+
*Hypophthalmichthys nobilis* (Hypo)	0.54	0.20	0.63	0.49	0.47	0.16	1.09	1.35
*Carassius auratus* (Cara)	+	+	+	+	+	+	+	+
*Cirrhinus molitorella* (Cirr)	2.88	21.48	9.52	6.55	6.50	4.96	4.36	7.10
*Ctenopharyngodon idella* (Cten)	1.29	0.56	1.45	0.72	1.04	0.52	1.05	1.23
*Cyprinus carpio* (Cypr)	0.11	0.05	0.11	0.05	0.02	0.00	0.01	0.02
*Discogobio tetrabarbatus* (Disc)	+	+	+	+	+	+	+	+
*Elopichthys bambusa* (Elop)	0.43	0.61	0.82	0.28	0.32	0.48	0.71	0.56
*Erythroculter dabryi* (Eryt)	+	+	+	+	+	+	+	+
*Chanodichthys recurviceps* (Chan)	+	+	+	+	+	+	0.59	+
*Garra orientalis* (Garr)	+	+	0.09	+	+	+	+	+
*Hemiculter leuscisculus* (Hemi)	3.57	12.59	4.72	2.67	3.93	6.42	4.24	3.28
*Hypophthalmichthys molitrix* (Hypop)	1.36	2.61	5.89	1.86	1.90	0.92	2.71	3.94
*Megalobrama terminalis* (Mega)	20.22	59.69	15.77	14.92	11.29	11.06	27.43	7.79
*Mylopharyngodon piceus* (Mylo)	0.05	1.13	0.29	0.32	0.35	0.04	0.10	0.29
*Ochtobius elongatus* (Ocht)	0.05	0.06	0.09	0.16	0.28	0.20	0.39	0.19
*Parabramis pekinensis* (Para)	0.89	3.58	1.90	0.63	0.79	0.47	0.59	0.76
*Pseudolaubuca engraulis* (Pseud)	+	+	+	+	+	+	+	+
*Pseudolaubuca sinensis* (Pseu)	1.43	2.65	3.06	0.84	0.42	0.33	0.53	0.49
*Barbodes semifasciolatus* (Barb)	+	+	+	+	+	+	+	+
*Metzia lineata* (metz)	+	+	+	+	+	+	+	+
*Rhodeus ocellatus* (Rhode)	+	+	+	+	+	+	+	+
*Rhodeus sinensis* (Rhod)	+	+	0.02	+	+	+	+	+
*Squalidus argentatus* (Squa)	1.02	0.91	3.92	2.01	4.87	2.19	1.05	19.16
*Squaliobarbus curriculus* (Squai)	23.92	60.58	66.10	37.78	29.84	23.25	49.99	59.09
*Xenocypris macrolepis* (Xenoc)	+	+	+	+	+	+	+	+
*Xenocypris davidi* (Xeno)	14.74	37.94	12.31	5.24	23.88	3.13	10.66	8.24
Cobitidae								
*Sinibotia robusta* (Sinib)	0.18	0.90	5.65	2.23	1.85	0.61	1.00	2.63
*Cobitis sinensis* (Cobi)	+	+	+	+	+	2.47	0.56	+
*Misgurnus anguillicaudatus* (Misg)	+	+	+	+	+	+	+	+
**CYPRINODONTIFORMES**								
Poeciliidae								
*Gambusia affinis* (Gamb)	+	0.06	+	+	+	+	+	+
**PERCIFORMES**								
Percichthyidae								
*Siniperca knerii* (Sinik)	+	+	+	+	+	+	+	+
*Siniperca scherzeri* (Sini)	0.26	0.21	0.44	0.16	0.16	0.04	0.14	0.16
Cichlidae								
*Oreochromis mossambicus* (Oreo)	0.004	0.011	0.021	+	0.003	+	0.018	0.019
Gobiidae								
*Rhinogobius giurinus* (Rhin)	1.39	1.66	1.08	2.90	0.49	0.69	2.05	0.63
Eleotridae								
*Eleotris oxycephala* (Eleo)	0.01	0.01	+	+	+	+	+	+
Channidae								
*Channa argus* (Chann)	+	+	+	+	+	+	+	+
*Channa maculate* (Chan)	+	+	+	+	+	+	+	+
*Mastacembelidae*								
*Mastacembelus armatus* (Mast)	+	+	+	+	+	+	+	+
**SALMONIFORMES**								
Salangidae								
*Salanx reevesii* (Sala)	1.08	1.01	0.64	0.54	0.13	0.45	0.19	0.31
**SILURIFORMES**								
Siluridae								
*Silurus asotus* (Silu)	+	+	0.03	+	+	+	0.35	0.01
Clariidae								
*Clarias fuscus* (Clar)	+	+	+	+	+	+	+	+
*Parabotia fasciatus* (Para)Siluridae	+	+	+	+	+	+	+	+
*Pterocryptis cochinchinensis* (Pter)	+	+	+	+	+	+	+	+
Bagridae								
*Tachysurus crassilabris* (Tachc)	+	+	+	+	+	+	+	+
*Tachysurus argentivittatus* (Tacha)	+	+	+	+	+	+	+	+
*Tachysurus virgatus* (Tachv)	+	+	+	+	+	+	+	+
*Hemibagrus macropterus* (Hemib)	+	+	+	+	+	+	+	+
*Tachysurus fulvidraco* (Tachf)	+	+	+	+	+	+	+	+
**SYNBRANCHIFORMES**								
Synbranchidae								
*Monopterus albus* (Mono)	+	+	+	+	+	+	+	+
N (×10^10^ ind)	22.47	56.36	52.26	31.19	26.74	16.23	46.51	61.76

### Interactions between Years and Seasons

The interaction between years and seasons were further explored using a two-factor orthogonal array analysis. Results indicated that the abundance of seven species namely, *Cirrhinus molitorella* (Cirr), *Ctenopharyngodon idella* (Cten), *Elopichthys bambusa* (Elop), *Ochtobius elongatus* (Ocht), *Oreochromis mossambicus* (Oreo), *Sinibotia robusta* (Sinib) and *Xenocypris davidi* (Xeno)) changed significantly over months, but not over years, with monthly changes being independent of yearly changes. Five species, (*Hypophthalmichthys nobilis* (Hypo), *Hypophthalmichthys molitrix* (Hypop), *S*. *reevesii* (Sala), *S*. *argentatus* (Squa) and *S*. *curriculus* (Squai)) changed significantly with each month, but not with years, but the monthly changes did interact with the years (Table 2). Two species, namely (*C*. *carpio* (Cypr) and *H*. *leuscisculus* (Hemi)) changed significantly both with months and years, but the monthly changes were independent of the years. Six species, (*M*. *terminalis* (Mega), *Mylopharyngodon piceus* (Mylo), *Parabramis pekinensis* (Para), *Pseudolaubuca sinensis* (Pseu), *Rhinogobius giurinus* (Rhin) and *Siniperca scherzeri* (Sini)) changed significantly both monthly and yearly; the monthly changes being dependent on annual changes (Table 2). These results show some species with obvious seasonal patterns and some of them with annual variability.

**Table 2:** The significance of observed differences between years and months in fish assemblage structure. * = P < 0.05; ** = P < 0.01; *** = P < 0.001

Species	Years	Months	Years × months
*Cirrhinus molitorella* (Cirr)		***	
*Ctenopharyngodon idella* (Cten)		***	
*Elopichthys bambusa* (Elop)		***	
*Ochtobius elongatus* (Ocht)		***	
*Oreochromis mossambicus* (Oreo)		*	
*Sinibotia robusta* (Sinib)		**	
*Xenocypris davidi* (Xeno)		**	
*Hypophthalmichthys nobilis* (Hypo)		***	***
*Hypophthalmichthys molitrix* (Hypop)		***	***
*Salanx reevesii* (Sala)		*	***
*Squalidus argentatus* (Squa)		*	***
*Squaliobarbus curriculus* (Squai)		***	**
*Cyprinus carpio* (Cypr)	**	**	
*Hemiculter leuscisculus* (Hemi)	***	***	
*Megalobrama terminalis* (Mega)	*	***	*
*Mylopharyngodon piceus* (Mylo)	**	***	***
*Parabramis pekinensis* (Para)	***	***	**
*Pseudolaubuca sinensis* (Pseu)	***	***	***
*Rhinogobius giurinus* (Rhin)	***	***	***
*Siniperca scherzeri* (Sini)	***	***	***

### Annual change occurrence patterns

**Fig 3:**
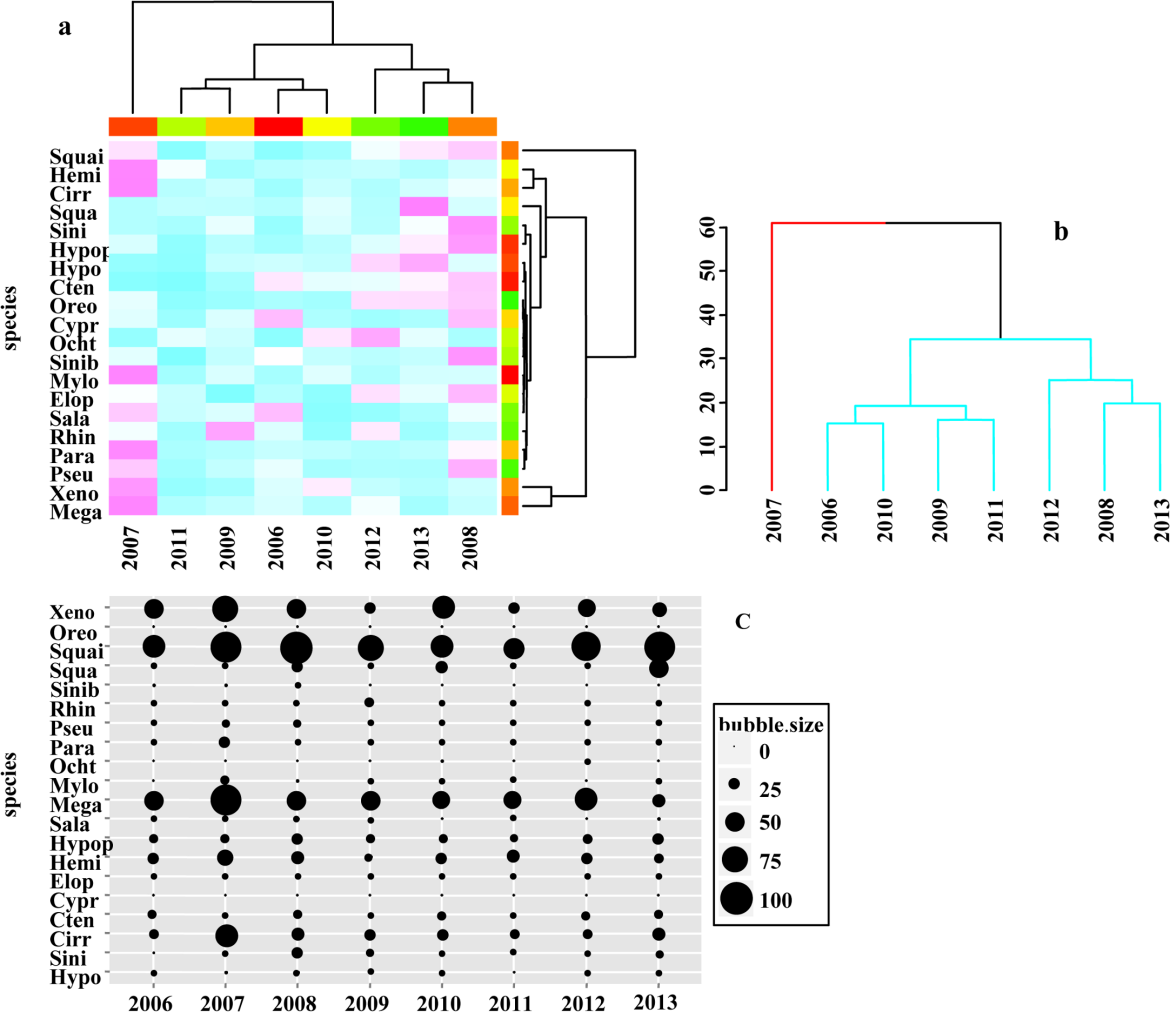
Annual temporal occurrence patterns of fish larvae from 2006 to 2013 in the Pearl River. (a) Heatmap showing the species and year clustering association for the fish larvae survey, with species assemblage on the x-axis and time classes on the y-axis. The colours, white (low ratio) to violet (high ratios), indicate the strength of association between species abundances and time variables. Weak correlations between variables are displayed in white and blue, while stronger correlations are shown in violet and pink. (b) The dendrogram produced by SIMPROF analysis. (c) Bubble plot of the fish larvae abundance, each bubble value represents the percentage of the maximum yearly larvae catch.

The yearly abundances of fish larvae showed some variability. In 2007, the dominant species were *S*. *curriculus* (Squai), *M*. *terminalis* (Mega), *X*. *davidi* (Xeno), *C*. *molitorella* (Cirr) and *H*. *leucisculus* (Hemi) with high abundances recorded. The 2008, 2013 and 2012 grouping was dominated by three species namely, *S*.*curriculus* (Squai), *M*. *terminalis* (Mega) and *X*. *davidi* (Xeno) which showed relatively high abundance. In the 2006, 2009, 2010 and 2011 grouping, fish abundances were scarce overall; dominat speices were *C*. *carpio* (Cypr), *S*. *reevesii* (Sala) and *R*. *giurinus* (Rhin) (Fig 3A & 3C). The dendrogram produced by the SIMPROF analysis also showed that 2007 was significantly different from other years, while the other two groupings showed no significant difference (Fig 3B). These differences were also confirmed by the one-way ANOSIM analyses, which showed that the structure and composition of the fish assemblage displayed in 2007 was significantly different from other years (R_ANOSIM_ = 0.278; p < 0.05). Again, the differences amongst the other were not significant.

The bubble plot (Fig 3C) of the yearly average catches also confirmed that six species were numerically dominant namely, *S*. *curriculus* (Squai) as the most abundant species, followed by *M*. *terminalis* (Mega), *X*. *davidi* (Xeno), *C*. *molitorella* (Cirr), *H*. *leucisculus* (Hemi) and *S*. *argentatus* (Squa). From the annual bubble plot, it can be seen that five species numbers decreased from 2006 to 2013; these include *C*. *molitorella* (Cirr), *H*. *leucisculus* (Hemi), *S*. *reevesii* (Sala), *M*. *terminalis* (Mega) and *P*. *pekinensis* (Para). On the other hand three species populations increased, namely, *H*. *nobilis* (Hypo), *O*. *elongatus* (Ocht) and *S*. *argentatus* (Squa). All others showed no change during the study period.

### Seasonal occurrence patterns

**Fig 4:**
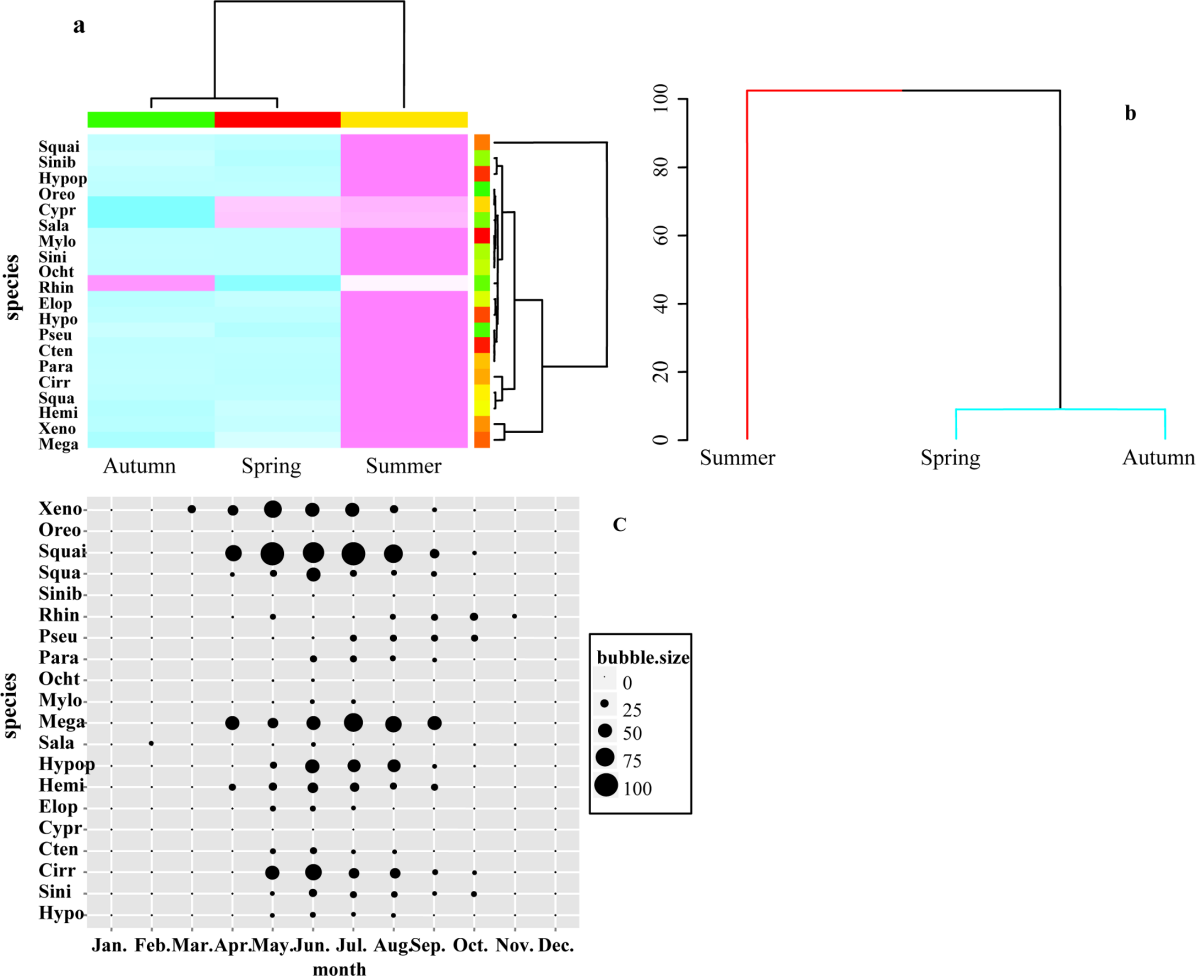
Seasonal occurrence patterns of fish larvae in the Pearl River from 2006 to 2013. Each month's data is the mean figure over the eight years of sampling. (a) Heatmap showing the ‘species–month’ association for fish larvae. (b) Dendrogram produced by SIMPROF analysis. (c)Bubble plot of monthly fish larval abundance; each bubble value is a percentage of the maximum catch over the year.

Fish larvae abundances showed apparent seasonal variability, with summer displaying the highest fish larvae abundance, while spring and autumn had low abundances overall (Fig 4A and 4B). Summer samples had a significantly different composition in comparison to the other seasons (R_ANOSIM_ = 0.52, P < 0.001). Spring and autumn showed no significant difference. The majority of larvae occur in the summer (between May and September, Fig 4A), peaking from June through August (Fig 4C). May and September are characterized by both early or late spawning. This may reflect abundant food sources in the summer period in the Pearl River. *C*. *carpio* (Cypr) and *S*. *reevesii* (Sala) larvae occurred mainly in the spring and *R*. *giurinus* (Rhin) larvae in the autumn (Fig 4A).

### Relationships between temporal patterns of larval fish and environmental factors

**Fig 5:**
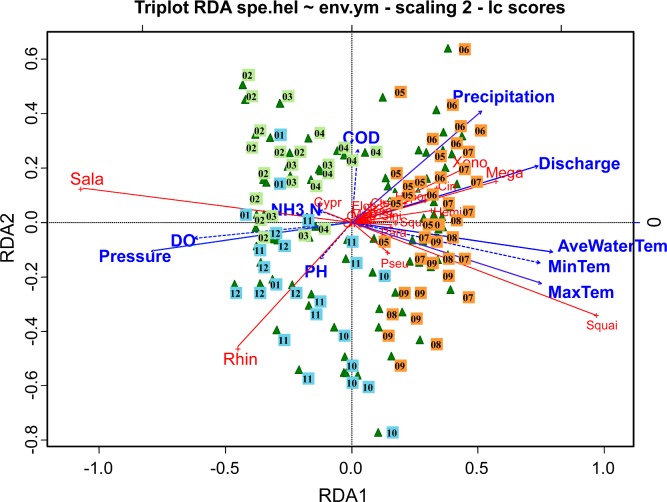
Redundancy analysis triplot showing relationships among fish larval abundances and environmental variables (scaling 2). RDA1 and RDA2 scales are related to the larval variables, while the top and right-hand scales are related to the environmental variables. Green triangles are the samples. Environmental variables are represented by blue arrows; solid lines depict significant environmental factors as opposed to dashed lines which are not significant. Red lines without arrows are species.

The RDA model revealed the relationships between larvae seasonal occurrence patterns and environmental factors (Fig 5). The combined effect of the first two canonical axes explain 91% of the total variance of the data, the first axis alone accounts for 79%. The unadjusted and adjusted R^2^ retrieved from the RDA results are 0.602 and 0.546 respectively, and the p value (ANOVA test) of the first two canonical axes was sufficiently low to denote a good sample separation along the axis. The eigenvalues and their contribution to variance are shown in Table 3.

**Table 3:** Summary of the RDA analysis. * = P < 0.05; *** = P < 0.001.

	RDA1	RDA2	RDA3	RDA4	RDA5	RDA6
F	90.7355	13.5599	5.9460	1.4228	1.2551	0.7565
p value	0.001***	0.001***	0.048*	0.194	0.292	0.591
Eigenvalue	0.2543	0.0379	0.0152	0.0045	0.0037	0.0022
Proportion explained	0.7933	0.1182	0.0475	0.0141	0.0114	0.0066
Cumulative Proportion	0.7933	0.9116	0.9590	0.9732	0.9846	0.9912
MaxTem	0.9057	-0.2715	-0.2410	-0.0838	-0.0248	-0.0519
MinTem	0.8993	-0.1790	-0.2855	-0.0563	-0.0947	0.0585
AveWaterTem	0.9587	-0.1307	-0.1312	-0.1532	-0.0561	0.0521
Precipitation	0.6375	0.4988	-0.2957	-0.0475	0.3518	-0.0609
Pressure	-0.9318	-0.1169	0.1562	0.2030	0.1279	0.0327
Discharge	0.8900	0.2499	0.0862	0.2980	-0.0402	-0.2039
pH	-0.1489	-0.1609	0.3416	-0.1589	-0.1830	-0.0343
DO	-0.7496	-0.0693	0.2769	0.1171	0.2448	0.3325
COD	0.0286	0.3235	0.0661	0.0254	0.2447	-0.0203
NH3.N	-0.1544	0.0531	-0.0234	0.0286	0.0542	0.2249

The RDA triplot (scaling = 2) shows that temperatures, river discharge, atmospheric pressure, as well as DO concentrations play important roles in the dispersion of the samples along the RDA1 axis (Fig 5). The second axis did not have characteristic variables, except for COD which showed a low contribution to this axis. The triplot shows a gradient from left to right organized in groups divided by RDA1 and RDA2. Overall, the progression was from low season characterized by high DO and atmospheric pressure, to summer with high temperature, precipitation and river discharge. The triplot shows obvious seasonal assemblage patterns of fish species correlated with different sets of explanatory variables: starting with a group comprised of spring samples. The abundance of *S*. *reevesii* (Sala) and *C*. *carpio* (Cypr), is high in spring, and is associated with higher DO concentrations, higher atmospheric pressure and lower temperature. *M*. *terminalis* (Mega), *X*. *davidi* (Xeno), and *C*. *molitorella* (Cirr) have similar occurrence patterns, all being associated with high precipitation, high river discharge and low pressure and low DO concentrations in summer. The abundance of *S*. *curriculus* (Squai) is also associated with summer, mostly in accordance with the highest temperatures experienced, and the lowest NH_3_–N values. *R*. *giurinus* (Rhin) abundance is associated with higher atmospheric pressure and DO concentrations, lower precipitation and lower river discharge rates in the autumn. *R*. *giurinus* (Rhin) lay their eggs in holes in the sand and need to avoid flow disturbance from high river discharge. Most other species are clustered together, separated from these extremes and show mostly shorter projections, indicating that they are more related to intermediate ecological conditions.

*X*. *davidi* (Xeno), *M*. *terminalis* (Mega) and *C*. *molitorella* (Cirr) together contributed 39% of the total number of larvae, mainly occurring in June and July. *S*. *curriculus* (Squai) was the most dominant species, which accounted for 41% of the total number of larvae, mainly occurring in July, August and September (Table 4). These dominant fish species stagger their spawning period, which avoids competition for food and other resources, and allows for coexistance. They avoid competition during early life stages.

**Table 4:** The percentage of species in the total fish larvae count.

Taxon	Percentage (%)
*Spring group*	0.90
Salanx reevesii (Sala) *	0.90
*Summer group*	39.21
Megalobrama terminalis (Mega) *	17.28
Xenocypris davidi (Xeno) *	12.87
Cirrhinus molitorella (Cirr) *	9.06
*Autumn group*	41.20
Squaliobarbus curriculus (Squai) *	41.20
*Winter group*	0.46
Rhinogobius giurinus (Rhin) *	0.46
*Intermediate group*	18.23
* Hypophthalmichthys nobilis*	*0*.*66*
* Ctenopharyngodon idella*	*1*.*02*
* Cyprinus carpio*	0.03
* Elopichthys bambusa*	0.44
* Hemiculter leuscisculus*	3.34
* Hypophthalmichthys molitrix*	2.33
* Mylopharyngodon piceus*	0.28
* Ochtobius elongatus*	0.16
* Parabramis pekinensis*	0.91
* Pseudolaubuca sinensis*	0.80
* Squaliobarbus curriculus*	5.58
* Siniperca scherzeri*	0.19
* Oreochromis mossambicus*	0.01
* Salanx reevesii*	0.38
* Sinibotia robusta*	2.10

* = species with apparent seasonal patterns

Permutation tests with 1000 iterations were performed and found that the model is very significant. The impact of climatic factors on the occurrence of larval fish is greater than the impact of small-scale environmental factors. Using the stepwise procedure, we selected the 5 best explanatory variables whose partial contribution optimally explains the largest portion of the variance of the response data by selecting the highest R^2^ if that variable is also significant (permutation test) at a preselected significance level. Results show that mean water temperature, discharge, atmospheric pressure, maximum temperature and precipitation explain the largest portion of variance (Fig 5). These variables were associated with horizontal axis 1 which explains 82% of the total variance. The importance of these five variables decrease in turn, respectively. The adjusted R^2^ result retrieved from the RDA model (constrained by these five important environmental variables) is 0.538. This means these five environmental variables can explain almost all the variables.

## Discussion

To date, there has been little research regarding the reproductive patterns of fishes in the Pearl River, which is essential information for fishery regulation and management. This research has shown that fish larvae in the lower reaches of the Pearl River are not randomly distributed in time, but show significant seasonal variability, with the greatest numbers occurring in the summer period, peaking in June and running through August. Temporal staggering was noted between the very abundant *M*. *terminalis* (Mega) and *S*. *curriculus* (Squai) larvae, both important commercial fish in the southern Chinese Pearl River. The occurrence of *S*. *curriculus* and *M*. *terminalis* climax in the summer; *S*. *curriculus* peaking in June and July, while *M*. *terminalis* overtakes (after a short overlap) the dominant position a little later in July, August and September. The larvae of both these fish species mainly feed on rotifers, copepod nauplii and cladocerans before their feeding habits diverge [45–46]; thus the prey of these two larvae most likely have some ephemeral, spatial overlap. These species however stagger their spawning bloom period which advantageously avoids direct competition for food and other resources during the early life stages. This suggests that the fish in the Pearl River show a coexistence strategy involving different temporal patterns of fish larval occurrence. Understanding processes such as this is the basis of wise conservation decisions and conservation of fish community diversity.

Recruitment is crucial for population survival [47]. Many species reproduce when conditions are most favorable for the survival of young [48]. In our study area, the abundance of phytoplankton and mesozooplankton showed a peak during the high temperature season [49–50], which coincides well with the peak abundance of total fish larvae in summer (from May to September). This confirms an essential aspect of fish reproduction i.e. that larvae be placed in favourable habitats with characteristics that will maximise the probability of survival through the planktonic phase. Favourable habitats have been defined mainly by high abundance of food at the right time [51].

Understanding the mechanisms that affect recruitment is critical to the effective conservation of wild fish populations [52]. Generally, the water temperature, river discharge and internal factors (e.g. circa-annual rhythm) are the main factors that regulate the spawning period in freshwater [53]. Existing research shows that temperature is crucial for fish breeding [54] and has a great influence on population dynamics [55] since temperature will stimulate the gonads [56–57] and change the spawning frequency of fish [58]. These findings are consistent with our results. The seasonal pattern of fish larval occurrence was mainly affected by water temperature in the Pearl River. It is generally accepted that temperature is among the main factors determining temporal changes in fish community structure and assemblage [59]. The seasonal pattern of fish larval occurrence was also dependent on seasonal flow rates in the Pearl river, with high peak flow rate during summer and low flows during spring and autumn. The reproduction of the riverine fishes is linked to specific flow events and flood pulses which appear to trigger spawning [60–61]. Hydrological changes are among the most important factors affecting regeneration and reproduction of fish populations, especially for fish with drifting eggs. It was also noted that atmospheric pressure is also a very important aspect in regulating the spawning activity of fishes in the study area. This may be due to the fact that the atmospheric pressure can affect the DO concentrations in the water, and thus affect the appetite and diet of breeding fish [62].

It was also noted that the occurrence of different fish larvae is directly associated with specific environmental requirements, especially where dominant species exist. *M*. *terminalis* (Mega) and *X*. *davidi* (Xeno) are strongly associated with high precipitation, high river discharge, low atmospheric pressure and DO concentrations in the summer months. Since these particular species all lay their sticky eggs onto the sand and rocks, they require specific complex hydrogeological conditions which will facilitate the adherence of their eggs. *S*. *curriculus* (Squai) showed the greatest ability to adapt to varied river discharge levels and associated with the highest temperatures and lowest NH_3_–N in our study area. *R*. *giurinus* (Rhin) on the other hand have different egg laying habits, laying their eggs in holes in the sand and therefore need to avoid flow disturbances such as high river discharges or flash floods. This too is important information for fisheries conservation.

It was hypothesized that fish larvae are sensitive to environmental perturbations, and it was anticipated that differences could be observed in occurrence patterns which were related to anomalously warm or cold conditions. Indeed, it was demonstrated that maximum and minimum air temperatures are strongly correlated to mean water temperatures (Fig 5), which have a strong impact on reproduction patterns. In particular, maximum air temperatures greatly affect the occurrence patterns of the most abundant species, *S*. *curriculus* (Squai). Although some water quality parameters, such as reduced oxygen during hot summers, could be a potential source of stress for fish, no parameters reached lethal levels [63]. The impact of climatic factors (such as temperature, river discharge, atmospheric pressure and precipitation) on the occurrence of fish larvae is greater than the impact of water quality factors in the Pearl river. This means that the occurrence of fish larvae is affected by regional climate first, and then by local environmental factors. This also validates the theory that climate influences a variety of ecological processes. Local weather parameters such as temperature drive temporally and spatially averaged exchanges of heat, that ultimately determine an organism's activities, such as growth and recruitment patterns [62].

Both globally and undeniably locally (in the Pearl River), fishery resources are decreasing. Inter-annual abundances of fish larvae showed some variability with numbers dropping from 56.36×10^10^ ind in 2007 to 16.23×10^10^ ind. in 2011. The obvious reason for this decline is the construction of the Changzhou Dam (located near the study area), at the end of 2007. The construction of the Changzhou Dam changed the continuum of the Pearl River, modifying the hydrology and flow, and consequently adversely affecting fish migration and reproduction [64–65]. Larval abundances decreased significantly post construction. Most economically important fish species in the Pearl River are affected by interannual river discharge variability [25], especially migratory fish species and species that cast their drifting eggs in running water, such as the large traditional domestic carps, namely, silver carp, bighead carp, grass carp and black carp. Fish larvae abundances have recovered to some degree since 2012. This is largely due to the implementation of a fishing moratorium in the Pearl River. Fishermen have not been allowed to fish during the period April 1st to June 1st since 2011. Since our research has been carried out both pre and post moratorium, it has influenced the inter-annual variability of fish larvae abundance. There is evidence that quantifiable relationships exist between hydrology and the density of young fish: high flows both before and during the spawning period have been positively correlated with recruitment [66–68]. Altering natural river flow patterns typically leads to a reduction in fisheries resources, in conjunction with a decline in the diversity of fish communities [69–72]. Spawning was delayed and rapidly suppressed in the middle reach of the Yangtze River, China post construction of the Three Gorges Dam [73]. This long-term monitoring of fish larval abundances and other environmental factors in the Pearl River are ongoing, and will build more comprehensive data in the future, to confidently identify and understand in greater detail how environmental factors might be influencing annual patterns in this valued river system.

Larval sampling surveys are effective in obtaining useful information on fish populations, compared to intense labour-induced capturing and analysing of mature fish populations. Trawlers used to capture adult fish have a serious tendency to damage resources, since large sweeping quantities of adult fish are captured, which overall is injurious to conservation. In this study, most species showed marked seasonality of larval occurrence, with high abundances in one particular period, although year-round occurrence was also observed. This indicates that fish larvae in the Pearl River have a species-specific pattern of temporal occurrence. The intense long-term, on-going, ecological research which was carried out three times a day every 2 days for several years is rarely found and reports approximate spawning periods of the adults in the Pearl River. These results have several implications for the conservation and the recovery of fisheries resources in the Pearl River. Conservation planning decisions and managements, such as an appropriate timetable for fishing moratoria can be established based on the spawning patterns outlined here. More information on larval feeding behavior and long-term monitoring are needed to adequately understand resource distribution inter-annual variability amongst the fish of the Pearl River. Information such as this will help clarify the mechanisms that would be required to maintain high abundance and species diversity in the near future.

## Supporting Information

S1 Table Data of experiment (Excel). The variables are self-evident. (XLSX)
