# Correction: Temporal Patterns of Larval Fish Occurrence in a Large Subtropical River

**DOI:** 10.1371/journal.pone.0156556

**Published:** 2016-05-26

**Authors:** Fangmin Shuai, Xinhui Li, Yuefei Li, Jie Li, Jiping Yang, Sovan Lek

There are errors in the Funding section. The correct funding information is as follows: This work was supported by 2013GXNSFEA053003 http://kjxm.gxsti.net Guangxi Natural Science Foundation of China (XL), 31400354 http://www.nsfc.gov.cn/ National Natural Science Foundation of China (FS), and 201303048 http://www.moa.gov.cn/ Special Fund for Agro-scientific Research in the Public Interest (XL).

Additionally, there is an error in the scale bar for Fig 1. Please see the corrected Fig 1 below.

**Fig 1:**
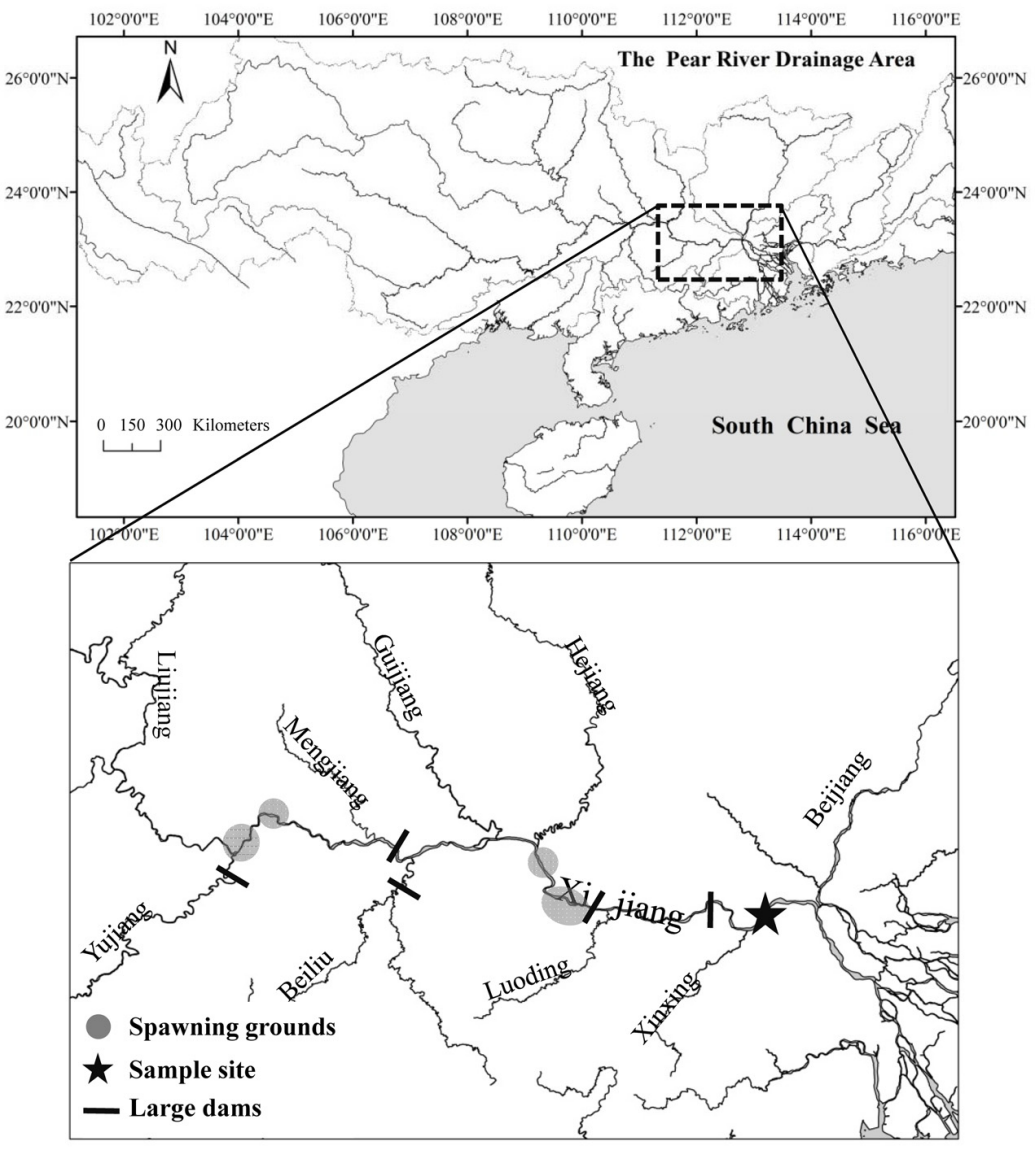
Study site.
